# Is there anything else to fish out of this “fishy” electrocardiogram?

**DOI:** 10.1007/s12471-022-01658-y

**Published:** 2022-01-13

**Authors:** A. Y. Andreou

**Affiliations:** 1 Department of Cardiology, Limassol General Hospital, Limassol, Cyprus; 2 University of Nicosia Medical School, Nicosia, Cyprus

Dear Editor,

The article by Devesa Neto et al. regarding the triangular QRS-ST‑T waveform (TW) pattern on an electrocardiogram (ECG), offers the opportunity to clarify the characteristics and prognostic implications of this ECG pattern in acute ST-segment elevation myocardial infarction (STEMI) [1].

The TW is characterised by an R wave with increased amplitude (≥ 1 mV), which merges with a steeply downsloping elevated ST segment, which in turn starts close to the peak of the QRS complex and the T wave. It is the same as the “giant R wave”, monophasic action potential-like and “shark fin-like” ECG waveforms [2]. Furthermore, a “triangulated” lambda-like QRS-ST‑T waveform seems to be a distinct, less pronounced form of the TW. Here, the steeply downsloping elevated ST segment begins at some point on the descending limb of the R wave but not below the quarter way point or close to the top of the R wave; it might denote less severe ischaemia.

Contrary to the authors’ statement, the “tombstone” ST-segment elevation waveform and TW are not the same, since the former comprises a convex upward sloping ST-segment elevation, which merges with a narrow R wave with minimal amplitude—if at all present—and the ascending limb of the T wave. The TW ECG pattern constitutes a rare manifestation of STEMI and is associated with acute, severe and extensive transmural ischaemia and consequently an increased risk of ventricular fibrillation/ventricular tachycardia, cardiogenic shock and in-hospital mortality [2]. It is commonly caused by culprit lesions in the left main or left anterior descending coronary artery.

Scrutiny of the ECG in question reveals TWs in V2–V3 expressing core ischaemia, lambda-like waveforms in V4–V5 expressing less severe ischaemia in the border zones surrounding the injured myocardial area, and signs of acquired complete right buddle branch block (RBBB; QR pattern in V1) and left posterior fascicular block (LPFB). The combination of new-onset RBBB and LPFB in acute anterior STEMI reflects extensive myocardial damage due to occlusion of the left anterior descending coronary artery proximal to the first septal perforator artery and concomitant severe disease in one or two additional coronary arteries.

This type of conduction disorder in acute MI is associated with increased risk of progression to complete atrioventricular block and increased mortality due to pump failure [3]. Furthermore, in the setting of acute coronary artery occlusion, cardiac arrest with an initial rhythm of pulseless electrical activity has been associated with pre-existing severe left ventricular systolic dysfunction [4]. Indeed, the just described clinical scenario reflects the clinical presentation and outcome of the patient in question, which could have been predicted by the presence of this conduction disorder rather than a TW ECG pattern. The patient’s outcome could have been better in the absence of the conduction disorder, since the TW ECG pattern has been associated with shockable ventricular arrhythmias showing a higher survival rate compared with pulseless electrical activity.

Electrocardiographic interpretation in acute STEMI should therefore not be limited to mere pattern recognition but needs to also include in-depth extraction of anatomical information with the aim of choosing the most effective therapeutic measures.
